# Prediction Model of Pathologic Central Lymph Node Negativity in cN0 Papillary Thyroid Carcinoma

**DOI:** 10.3389/fonc.2021.727984

**Published:** 2021-09-27

**Authors:** Xiujie Shu, Lingfeng Tang, Daixing Hu, Yuanyuan Wang, Ping Yu, Zhixin Yang, Chang Deng, Denghui Wang, Xinliang Su

**Affiliations:** ^1^ Department of Endocrinology and Breast Surgery, The First Affiliated Hospital of Chongqing Medical University, Chongqing, China; ^2^ Department of Breast and Thyroid Surgery, The Second Affiliated Hospital of Chongqing Medical University, Chongqing, China

**Keywords:** papillary thyroid carcinoma, nomogram, pathologic lymph node negativity, prognosis, central lymph node

## Abstract

**Background:**

Most patients with papillary thyroid carcinoma (PTC) have an excellent prognosis. Although central lymph node invasion is frequent, management *via* central lymph node dissection (CLND) remains controversial. The present study retrospectively investigated independent predictors of pathologic central lymph node negativity (pCLN-) and established a prediction model for pCLN- in clinical lymph node negativity (cN0) PTC.

**Methods:**

A total of 2,687 patients underwent thyroid surgery for cN0 PTC from 2013 to 2018 at the First Affiliated Hospital of Chongqing Medical University, and lobectomy plus ipsilateral CLND was the basic surgical extent. Clinicopathological characteristics were reviewed and analyzed. Univariate and multivariate analyses were performed to identify factors related to pCLN-. A prediction model was established based on the results of multivariate analyses.

**Results:**

The pCLN- rate was 51.5% (1,383/2,687). Multivariate analysis revealed that sex, age, thyroid stimulating hormone (TSH), size, location, laterality, unifocality and extrathyroidal extension negativity (ETE-) were independent predictors of pCLN-. The nomogram showed good discriminative ability (C-index: 0.784 and 0.787 in derivation and validation groups, respectively) and was well calibrated. We quantified the clinical usefulness of the nomogram by decision curve analysis. The median length of follow-up was 30 (range 12– 83) months, and 190 cases were lost, with a follow-up rate of 92.9% (2,497/2,687). Of the 2,687 patients included, 21 (0.8%) experienced recurrence.

**Conclusion:**

This nomogram, which integrates available preoperative clinicopathological features and intraoperative frozen biopsy outcomes, is a reliable tool with high accuracy to predict pCLN- in cN0 PTC.

## Introduction

Thyroid cancer is the most common endocrine malignancy, and papillary thyroid carcinoma (PTC) accounts for more than 90% of cases. The development of ultrasound and fine needle aspiration cytology rapidly improved the diagnosis of PTC in recent years (1–3). Most of PTCs are indolent and carry an excellent prognosis with low mortality. However, early-stage metastasis in cervical lymph nodes may occur, especially in the central compartment (Level VI), which is bound superiorly by the hyoid bone, inferiorly by the innominate artery, and laterally by the common carotid artery. Overall, accurate evaluation of lymph node status is of great significance for PTC treatment, TNM staging and risk stratification, but current methods for evaluating central lymph node (CLN) status have several limitations. Inconsistencies between clinical and pathologic evaluations may also exist. According to the 8th edition of the American Joint Committee on Cancer (AJCC) TNM staging, N0 is subdivided into N0a and N0b. N0a, also known as pN0, refers to negative lymph nodes confirmed by cytology or histology, and N0b, also known as cN0, indicates no metastatic lymph nodes (normal lymph nodes or nonspecific lymphadenitis) found on clinical and imaging examinations (4–7). Ultrasound is the most commonly used tool for the clinical evaluation of lymph nodes. The ultrasonic signs of typical lymph node metastasis primarily include a round shape, disappearance of the lymphatic hilum, mass hyperechoic regions, calcification foci (primarily microcalcification; coarse calcification is rare), cystic changes, and peripheral or mixed blood flow distribution signals. None of these features alone is sufficient to diagnose all metastatic lymph nodes, and the accuracy of the evaluation is limited based on considering the experience of clinicians, the function of instruments and heterogeneity among patients, the accuracy of the evaluation is limited (8–10). Therefore, only some patients with cN0 PTC have occult central lymph node metastasis (CLNM). The 2015 American Thyroid Association (ATA) and 2021 National Comprehensive Cancer Network (NCCN) guidelines recommend that T1 and T2 cN0 PTC should not be treated using prophylactic central lymph node dissection (CLND). However, surgical management of cN0 PTC remains controversial because of the presence of occult metastatic lymph nodes in cN0 PTC and the debate surrounding the effect of these lymph nodes on recurrence and prognosis (5, 11–14). Balancing the risks and benefits of lymph node dissection is crucial for individualized and precise treatment. For cN0 PTC with benign CLNs, it is reasonable to avoid prophylactic CLND, which protects the cervical tissue, reduces the extent of surgery and the corresponding complications, and facilitates the recovery of patients after surgery. This approach essentially promotes the development of scarless endoscopic thyroidectomy (SET). However, there have been few studies on the clinicopathological characteristics of these patients and a practical quantitative tool is urgently needed to identify these patients. We aim to establish a prediction model of pathologic central lymph node negativity (pCLN-) in patients with cN0 PTC.

## Materials and Methods

### Patient Collection

**Figure 1:**
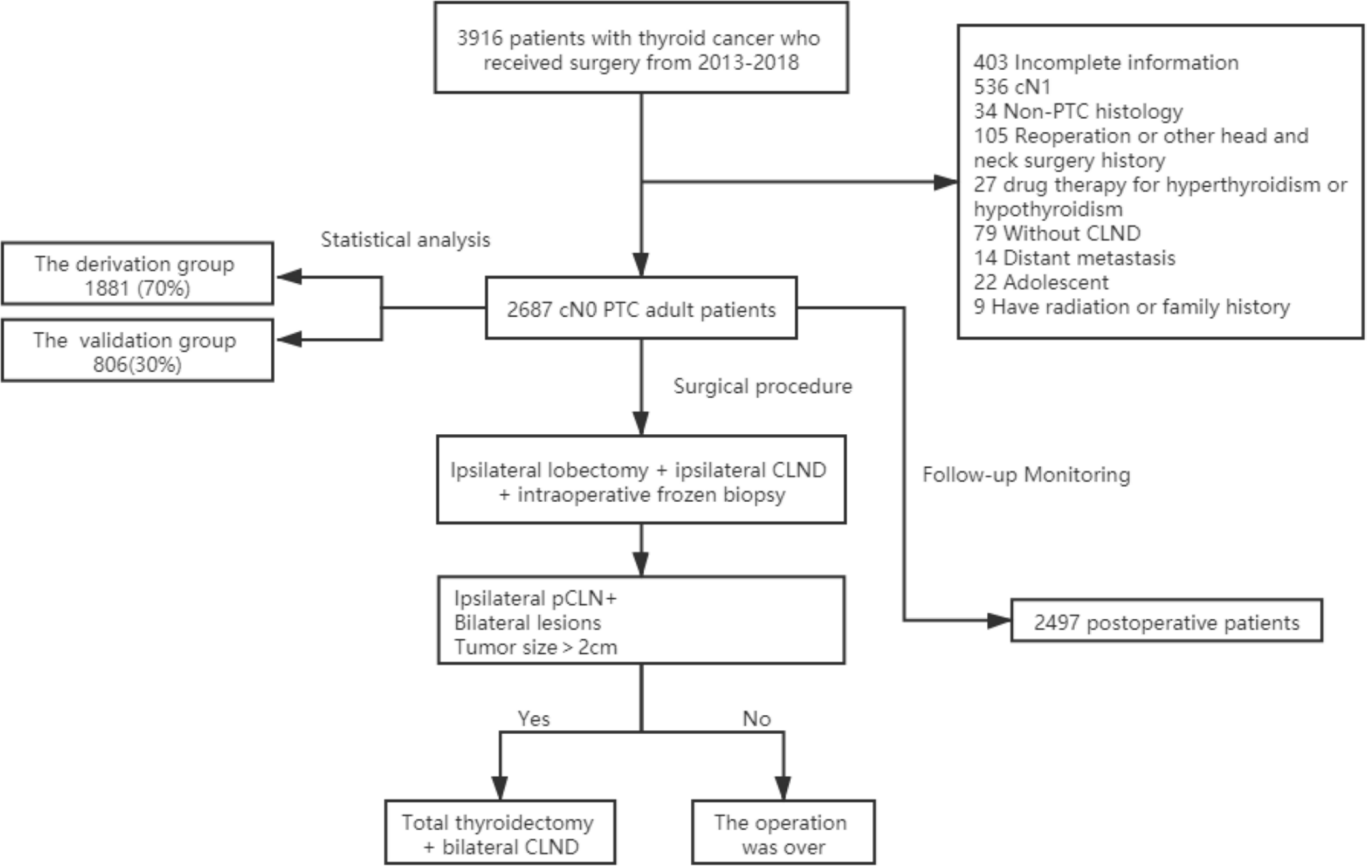
Flowchart of this retrospective study.

The local institutional ethics committee approved this retrospective study. Because there was no patient interest or privacy, informed consent was waived after careful review by the ethics committee. A total of 3,916 patients underwent surgery for thyroid diseases at the Department of Endocrine and Breast Surgery, The First Affiliated Hospital of Chongqing Medical University, between January 2013 and December 2018. All patients underwent at least a physical examination and cervical ultrasound examination before surgery. We used the cN0 assessment criteria of Kouvaraki et al. (1). Physical examination did not address enlarged lymph nodes or swollen lymph nodes that had a soft texture (2). Ultrasound examination showed no enlarged lymph nodes or swollen lymph nodes were oval and flat, regularly shaped, with clear boundaries between the cortex and medulla or a clear central fat hilum and no obvious malignant signs (15). Patients with any of the following conditions were excluded: incomplete clinicopathological data (403), CN1 confirmed by preoperative examination (536), non-PTC histology (34), reoperation or other head and neck surgery history (105), drug therapy for hyperthyroidism or hypothyroidism (27), absence of CLND (79), distant metastasis (14), adolescent (22), radiation exposure or family history of PTC (9). A total of 2,687 eligible patients were ultimately included in this study **(** **Figure 1** **)**.

### Surgical Procedure

For all patients enrolled, lobectomy plus ipsilateral CLND was the basic surgical treatment. Total thyroidectomy plus bilateral CLND was further performed for cases with bilateral tumors, tumors with a maximum diameter > 2 cm or intraoperative frozen section biopsy revealing CLNM. We routinely performed CLN intraoperative frozen section biopsy. Three pathologists blindly and independently diagnosed all resected thyroid and lymph node specimens.

### Demographic and Clinicopathological Data

Data for sex, age, body mass index (BMI), thyroid stimulating hormone (TSH), size, location, unifocality, laterality, Hashimoto’s thyroiditis (HT) and extrathyroidal extension negativity (ETE-) were collected and analyzed. The diameter of the largest lesion was recorded as the size, and the central location of the largest lesion was recorded as the location. When the tumor occupied almost the entire lobe, its location was recorded as “whole”. Notably, ipsilateral lesions indicated the presence of laterality, and the presence of only one lesion in one lobe indicated unifocality. Tumors in which growth was confined to the thyroid gland without capsule invasion were defined as ETE-, minimal ETE confirmed by intraoperative frozen section biopsy was not considered ETE-.

### Follow-up Monitoring

For all patients, the first follow-up was 1 month after surgery, and evaluations included a physical examination and measurement of serum TSH levels. Measurements of serum thyroglobulin (Tg) and Tg antibody levels were only performed for patients who underwent total thyroidectomy. The second follow-up was 6 months postoperatively. Cervical ultrasound examination was also performed for the first time. Regular evaluations were performed every 6-12 months thereafter. Patients with clinical evidence of recurrence, an unstimulated Tg level ≥2 ng/mL, a stimulated Tg level ≥ 20 ng/mL or serial increases in serum Tg (with negative Tg antibody), or an imaging abnormality were suspected of recurrence (16, 17). We performed fine needle aspiration in these patients and reoperations to identify the pathology of the recurrence.

### Statistical Analysis

The Mann-Whitney test was performed to compare differences in nonnormally distributed continuous variables (e.g., age, BMI, TSH, and size) between the two groups. Continuous variables with significant differences were converted into categorical variables. The chi-square test or Fisher’s exact test was used to compare categorical variables. The Excel random function was used to assign values to each patient, and the patients corresponding to these values were randomly sorted. Therefore, a total of 2,687 patients were randomly divided into a derivation group (70%) and a validation group (30%). Multivariate analyses were performed using logistic regression analysis to estimate the statistical significance of the relationships between pCLN- and the clinicopathological characteristics in the derivation group. P < 0.05 was considered a statistically significant difference. A nomogram was developed based on the results of these analyses, and the predictive ability of the nomogram was measured using the receiver operating characteristic (ROC) curve, concordance index (C-index) and calibration plot. To evaluate the clinical usefulness of the nomogram, decision curve analysis (DCA) was performed to calculate the net benefits at different threshold probabilities. The Kaplan-Meier method was used to estimate disease-free survival(DFS) rates. All statistical analyses were performed using SPSS version 25.0 (IBM Corp., USA), and graphs were generated using R software.

## Results

### Patient Characteristics

A total of 2,687 patients were included in the analysis (684 males and 2,003 females). The median age at diagnosis was 43 years (range 18-85 years). The median BMI level was 23.07 (range 15.56-36.98), and the mean serum concentration of TSH was 2.23 μIU/mL (range 0.01-97.18 μIU/mL). The median tumor size was 9 mm (range 3-64 mm). The numbers of tumors located in the upper portion, middle portion, lower portion, isthmus and whole thyroid were 592 (22.0%), 1,133 (42.2%), 806 (30.0%), 85 (3.2%) and 71 (2.6%), respectively. Right, left and bilateral tumors were detected in 1,211 (45.1%), 1,059 (39.4%), and 417 (15.5%) patients, respectively. Unifocality and ETE- were detected in 2,364 (88.0%) and 2,420 (90.1%) cases, respectively. Histological HT was present in 531 patients (19.8%) **(** **Table 1** **)**.

**Table 1:** Clinicopathological characteristics of all patients (derivation + validation groups).

Characteristics	Total		pCLN-		pCLN+		*P value*
	2687		1383		1304		-
Sex							<0.001
Male	684	25.5%	283	20.5%	401	30.8%	
Female	2003	74.5%	1100	79.5%	903	69.2%	
Age Years, Median (IQR)	43 (18)		45 (17)		41 (17)		<0.001
(18,25]	144	5.4%	48	3.5%	96	7.4%	
(25,35]	654	24.3%	269	19.5%	385	29.5%	
(35,45]	744	27.7%	375	27.1%	369	28.3%	
(45,55]	722	26.9%	428	30.9%	294	22.5%	
(55,65]	316	11.8%	192	13.9%	124	9.5%	
>65	107	4.0%	71	5.1%	36	2.8%	
BMI [Median (IQR)]	23.07 (4.43)		22.89 (4.49)		23.34 (4.41)		0.039
<18.5	121	4.5%	58	4.2%	63	4.8%	
[18.5,23)	1189	44.3%	649	46.9%	540	41.4%	
[23,25)	607	22.6%	296	21.4%	311	23.8%	
≥25	770	28.7%	380	27.5%	390	29.9%	
TSH μIU/mL, Median (IQR)	2.23 (1.96)		2.12 (1.92)		2.35 (1.97)		0.005
≤1	319	11.9%	178	12.9%	141	10.8%	
(1,2]	837	31.1%	466	33.7%	371	28.5%	
(2,3]	679	25.3%	333	24.1%	346	26.5%	
(3,4]	414	15.4%	188	13.6%	226	17.3%	
(4,5]	204	7.6%	99	7.2%	105	8.1%	
>5	234	8.7%	119	8.6%	115	8.8%	
Size mm, Median (IQR)	9 (8)		8 (5)		12 (10)		<0.001
≤5	337	12.5%	242	17.5%	95	7.3%	
(5,10]	1266	47.1%	775	56.0%	491	37.7%	
(10,15]	527	19.6%	206	14.9%	321	24.6%	
(15,20]	261	9.7%	86	6.2%	175	13.4%	
(20,25]	116	4.3%	39	2.8%	77	5.9%	
(25,30]	71	2.6%	15	1.1%	56	4.3%	
>30	109	4.1%	20	1.4%	89	6.8%	
Location							<0.001
Upper	592	22.0%	267	19.3%	325	24.9%	
Middle	1133	42.2%	762	55.1%	371	28.5%	
Lower	806	30.0%	313	22.6%	493	37.8%	
Isthmus	85	3.2%	29	2.1%	56	4.3%	
Whole	71	2.6%	12	0.9%	59	4.5%	
Laterality							<0.001
Left	1059	39.4%	583	42.2%	476	36.5%	
Right	1211	45.1%	644	46.6%	567	43.5%	
Bilateral	417	15.5%	156	11.3%	261	20.0%	
Unifocality							<0.001
No	323	12.0%	83	6.0%	240	18.4%	
Yes	2364	88.0%	1300	94.0%	1064	81.6%	
ETE-							<0.001
No	267	9.9%	72	5.2%	195	15.0%	
Yes	2420	90.1%	1311	94.8%	1109	85.0%	
HT							0.367
No	2156	80.2%	1119	80.9%	1037	79.5%	
Yes	531	19.8%	264	19.1%	267	20.5%	
CLND extent							<0.001
Ipsilateral	1606	59.8%	1041	75.3%	565	43.3%	
Bilateral	1081	40.2%	342	24.7%	739	56.7%	
CLNY, Median (IQR)	9 (8)		8 (8)		10 (9)		<0.001
CLNR, Median (IQR)	0.0 (0.3)		0.0 (0.0)		0.3 (0.3)		<0.001

PCLN, Pathologic central lymph nodes negative; PCLN+, Pathologic central lymph nodes positive; HT, Hashimoto’s Thyroiditis; CLNY, central lymph node yield; CLNR, central lymph node ratio.

### Lymph Node Yield and Lymph Node Ratio

Overall, the rate of pCLN- was 51.5%. The median central lymph node yield (CLNY), central lymph node ratio (CLNR), and the number of ipsilateral CLNDs in the pCLN- cohorts, were significantly lower than the pCLN+ cohorts **(** **Table 1** **)**. There were 1606 (59.8%) ipsilateral CLNDs and 1081 (40.2%) bilateral CLNDs. The median (IQR) CLNY and CLNR in ipsilateral CLNDs were 7 (7) and 0 (0.2), respectively, and medians in bilateral CLNDs were 11 (9.5) and 0.18 (0.43), respectively. (not shown in tables).

### Clinicopathological Characteristics of Patients in the Derivation Group

There were no significant differences in CLN pathology, sex, age, BMI, TSH, size, location, unifocality, laterality, ETE-, HT, CLND extent, CLNY or CLNR between the derivation and validation groups **(** **Table 2** **)**. We found significant differences in sex, age, BMI, TSH, size, location, unifocality, laterality, and ETE- between the pCLN- and pCLN+ cohorts in the derivation group based on univariate analysis. However, no significant difference was observed for HT (P=0.687). The number of CLNDs was excluded from our analysis due to collinearity.

**Table 2:** Difference between the derivation and validation data groups.

Characteristics	Total	Derivation Group		Validation Group		P Value
	2687	1881		806		-
Pathology						0.407
pCLN-	1383	978	52.0%	405	50.2%	
pCLN+	1304	903	48.0%	401	49.8%	
Sex						0.712
Male	684	475	25.3%	209	25.9%	
Female	2003	1406	74.7%	597	74.1%	
Age Years, Median (IQR)	43 (18)	43 (18)		43 (18)		0.493
(18,25]	144	103	5.5%	41	5.1%	
(25,35]	654	448	23.8%	206	25.6%	
(35,45]	744	533	28.3%	211	26.2%	
(45,55]	722	491	26.1%	231	28.7%	
(55,65]	316	230	12.2%	86	10.7%	
>65	107	76	4.0%	31	3.8%	
BMI [Median (IQR)]	23.07 (4.43)	23.05 (4.30)		23.14 (4.77)		0.515
>18.5	121	80	4.3%	41	5.1%	
[18.5,23)	1189	842	44.8%	347	43.1%	
[23,25)	607	431	22.9%	176	21.8%	
≥25	770	528	28.1%	242	30.0%	
TSH μIU/mL, Median (IQR)	2.23 (1.96)	2.24 (1.95)		2.22 (2)		0.287
≤1	319	228	12.1%	91	11.3%	
(1,2]	837	580	30.8%	257	31.9%	
(2,3]	679	476	25.3%	203	25.2%	
(3,4]	414	305	16.2%	109	13.5%	
(4,5]	204	132	7.0%	72	8.9%	
>5	234	160	8.5%	74	9.2%	
Size mm, Median (IQR)	9 (8)	9 (7)		10 (8)		0.781
≤5	337	240	12.8%	97	12.0%	
(5,10]	1266	883	46.9%	383	47.5%	
(10,15]	527	380	20.2%	147	18.2%	
(15,20]	261	177	9.4%	84	10.4%	
(20,25]	116	77	4.1%	39	4.8%	
(25,30]	71	47	2.5%	24	3.0%	
>30	109	77	4.1%	32	4.0%	
Location						0.07
Upper	592	411	21.9%	181	22.5%	
Middle	1133	822	43.7%	311	38.6%	
Lower	806	536	28.5%	270	33.5%	
Isthmus	85	60	3.2%	25	3.1%	
Whole	71	52	2.8%	19	2.4%	
Laterality						0.125
Left	1059	765	40.7%	294	36.5%	
Right	1211	830	44.1%	381	47.3%	
Bilateral	417	286	15.2%	131	16.3%	
Unifocality						0.709
No	323	229	12.2%	94	11.7%	
Yes	2364	1652	87.8%	712	88.3%	
ETE-						0.99
No	267	187	9.9%	80	9.9%	
Yes	2420	1694	90.1%	726	90.1%	
HT						0.651
No	2156	1505	80.0%	651	80.8%	
Yes	531	376	20.0%	155	19.2%	
CLND extent						0.151
Ipsilateral	1606	1141	60.7%	465	57.7%	
Bilateral	1081	740	39.3%	341	42.3%	
CLNY, Median (IQR)	9 (8)	8 (8)		9 (8)		0.445
CLNR, Median (IQR)	0.0 (0.3)	0.0 (0.3)		0.0 (0.3)		0.417

Multivariate analysis indicated that age (P < 0.001), sex (P < 0.001), TSH (P = 0.003), size (P < 0.001), location (P < 0.001), laterality (P = 0.005), unifocality (P < 0.001) and ETE- (P < 0.001) were independent predictors of pCLN-. Overall, BMI had little effect on pCLN-, with no statistical significance was found (P= 0.781) **(** **Table 3** **)**.

**Table 3:** Univariate and multivariate analysis of pCLN- in the derivation group.

Characteristics	Total		pCLN-		pCLN+		Univariate analysis	P Value	Multivariate analysis	P Value
	1881		978		903		OR (95% CI)		OR (95% CI)	
Sex								<0.001		<0.001
Male	475	25.3%	193	19.7%	282	31.2%	1 (reference)		1 (reference)	
Female	1406	74.7%	785	80.3%	621	68.8%	1.847 (1.495-2.281)		2.069 (1.608-2.663)	
Age Years, Median (IQR)	43 (18)		45 (16)		41 (17)			<0.001		<0.001
(18,25]	103	5.5%	40	4.1%	63	7.0%	1 (reference)		1 (reference)	
(25,35]	448	23.8%	184	18.8%	264	29.2%	1.098 (0.708-1.702)		0	
(35,45]	533	28.3%	271	27.7%	262	29.0%	1.629 (1.059-2.507)		0.884 (0.534-1.465)	
(45,55]	491	26.1%	289	29.6%	202	22.4%	2.253 (1.458-3.482)		1.212 (0.735-2.000)	
(55,65]	230	12.2%	143	14.6%	87	9.6%	2.589 (1.606-4.173)		2.074 (1.251-3.440)	
>65	76	4.0%	51	5.2%	25	2.8%	3.213 (1.726-5.98)		2.360 (1.357-4.104)	
BMI Median (IQR)	23.05 (4.30)		22.86 (4.27)		23.37 (4.17)			0.041		0.781
<18.5	80	4.3%	46	4.7%	34	3.8%	1 (reference)		1 (reference)	
[18.5,23)	842	44.8%	464	47.4%	378	41.9%	0.907 (0.571-1.442)		1.052 (0.626-1.766)	
[23,25)	431	22.9%	213	21.8%	218	24.1%	0.722 (0.446-1.169)		0.909 (0.524-1.577)	
≥25	528	28.1%	255	26.1%	273	30.2%	0.690 (0.429-1.11)		0.991 (0.574-1.712)	
TSH μIU/mL, Median (IQR)	2.24 (1.95)		2.12 (1.90)		2.36 (1.99)			0.012		0.003
≤1	228	12.1%	129	13.2%	99	11.0%	1 (reference)		1 (reference)	
(1,2]	580	30.8%	330	33.7%	250	27.7%	1.013 (0.744-1.380)		0.931 (0.654-1.325)	
(2,3]	476	25.3%	235	24.0%	241	26.7%	0.748 (0.545-1.028)		0.686 (0.477-0.986)	
(3,4]	305	16.2%	139	14.2%	166	18.4%	0.643 (0.455-0.908)		0.526 (0.353-0.783)	
(4,5]	132	7.0%	64	6.5%	68	7.5%	0.722 (0.470-1.11)		0.599 (0.365-0.983)	
>5	160	8.5%	81	8.3%	79	8.7%	0.787 (0.524-1.181)		0.649 (0.405-1.04)	
Size mm, Median (IQR)	9 (7)		8 (5)		12 (9)			<0.001		<0.001
≤5	240	12.8%	176	18.0%	64	7.1%	1 (reference)		1 (reference)	
(5,10]	883	46.9%	538	55.0%	345	38.2%	0.567 (0.413-0.778)		0.544 (0.385-0.768)	
(10,15]	380	20.2%	151	15.4%	229	25.4%	0.240 (0.169-0.341)		0.255 (0.173-0.376)	
(15,20]	177	9.4%	58	5.9%	119	13.2%	0.177 (0.116-0.271)		0.217 (0.136-0.345)	
(20,25]	77	4.1%	28	2.9%	49	5.4%	0.208 (0.120-0.358)		0.201 (0.110-0.370)	
(25,30]	47	2.5%	11	1.1%	36	4.0%	0.111 (0.053-0.231)		0.117 (0.053-0.258)	
>30	77	4.1%	16	1.6%	61	6.8%	0.095 (0.051-0.177)		0.187 (0.074-0.474)	
Location								<0.001		<0.001
Upper	411	21.9%	189	19.3%	222	24.6%	0.419 (0.329-0.534)		0.455 (0.348-0.595)	
Middle	822	43.7%	551	56.3%	271	30.0%	1 (reference)		1 (reference)	
Lower	536	28.5%	208	21.3%	328	36.3%	0.312 (0.249-0.391)		0.304 (0.236-0.392)	
Isthmus	60	3.2%	21	2.1%	39	4.3%	0.265 (0.153-0.459)		0.337 (0.179-0.635)	
Whole	52	2.8%	9	0.9%	43	4.8%	0.103 (0.049-0.214)		0.219 (0.071-0.671)	
Laterality								<0.001		0.005
Left	765	40.7%	417	42.6%	348	38.5%	1 (reference)		1 (reference)	
Right	830	44.1%	461	47.1%	369	40.9%	1.043 (0.856-1.270)		1.082 (0.864-1.356)	
Bilateral	286	15.2%	100	10.2%	186	20.6%	0.449 (0.339-0.595)		0.629 (0.452-0.875)	
Unifocality								<0.001		<0.001
No	229	12.2%	58	5.9%	171	18.9%	0.270 (0.197-0.369)		0.294 (0.206-0.419)	
Yes	1652	87.8%	920	94.1%	732	81.1%	1 (reference)		1 (reference)	
ETE-								<0.001		<0.001
No	187	9.9%	54	5.5%	133	14.7%	1 (reference)		1 (reference)	
Yes	1694	90.1%	924	94.5%	770	85.3%	2.956 (2.124-4.113)		2.235 (1.520-3.288)	
HT								0.687		–
No	1505	80.0%	786	80.4%	719	79.6%	1 (reference)			
Yes	376	20.0%	192	19.6%	184	20.4%	0.955 (0.761-1.197)			

### Prediction Models

Based on multivariate logistic regression analysis of the derivation group, we developed a prediction model for pCLN-.


Model:sex+age+TSH+size+location+laterality+unifocality+ETE−

**Figure 2:**
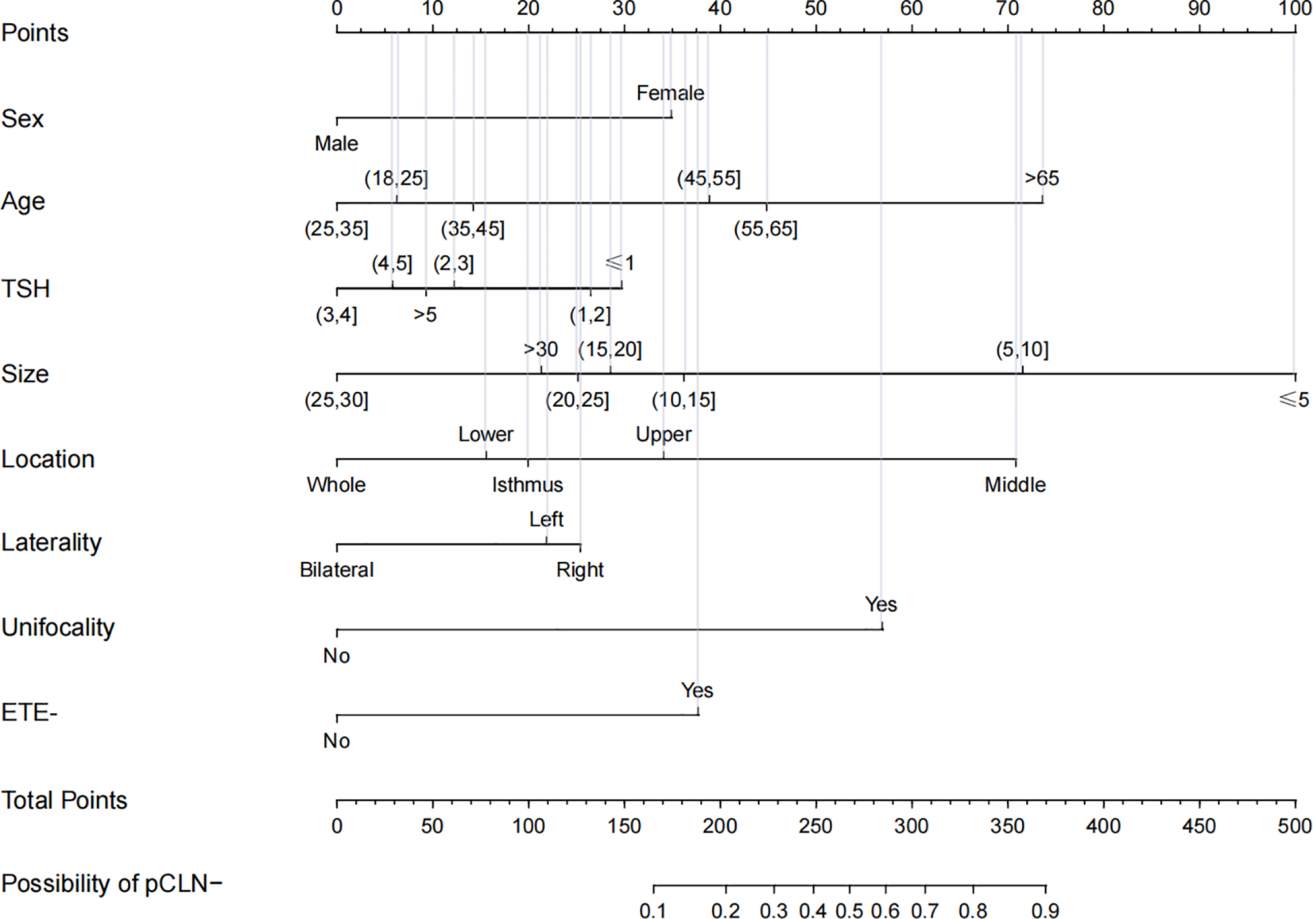
Nomogram for prediction of pCLN-. A line is drawn straight up to the point axis that corresponds with each patient variable to obtain the points. The sum of these points is located on the total score points axis. A line is drawn downwards to the risk axis to determine the possibility of pCLN-.

The nomogram shows the score of each variable on each scale. Therefore, the probability of pCLN- is determined by the total score of all variables. Inputting the necessary clinicopathological data concisely estimated the probability of pCLN- **(** **Figure 2** **)**.

**Figure 3:**
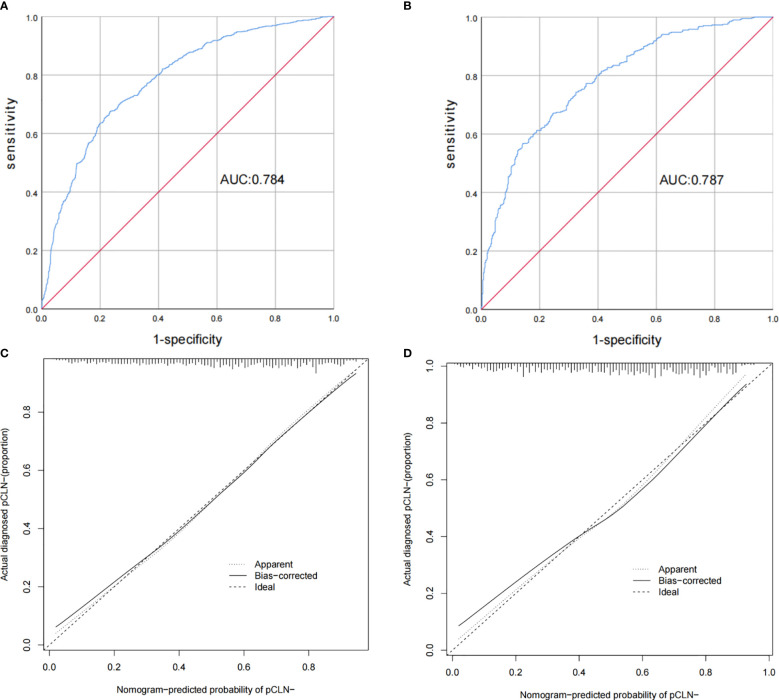
Receiver operating characteristic curves and calibration plot of the model. In the derivation group **(A)**, the sensitivity and specificity were 67.5% and 76.6%, respectively, with an AUC of 0.784 (95% CI 0.763–0.804). In the validation group **(B)**, the sensitivity and specificity were 67.2% and 75.3%, respectively, with an AUC of 0.787 (95% CI 0.756–0.818). The calibration plot depicts the calibration of the model in terms of the agreement between the predicted possibility of pCLN- and the observed outcomes of pCLN-. The derivation group **(C)** and the validation group **(D)** indicated good prediction.

**Figure 4:**
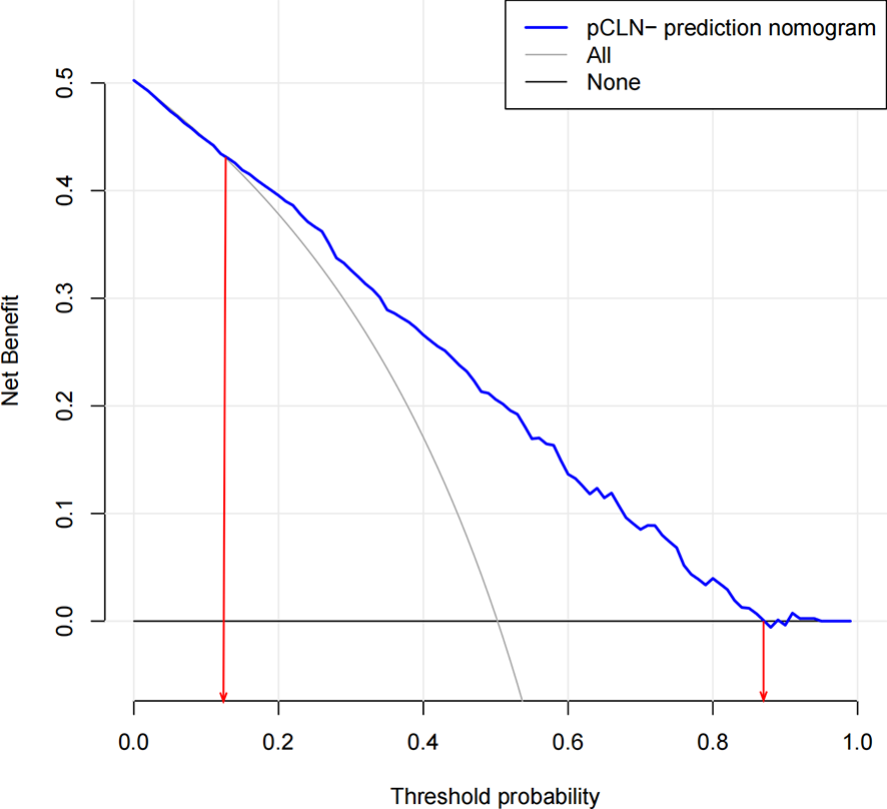
Decision curve analysis for the nomogram(in the validation group). The possibility of an individual is denoted as Pi. When Pi reaches a certain threshold (denoted as Pt), it is defined as positive, and intervention (not performing CLND) may be taken. Therefore, there are benefits (pros) of intervention for patients with pCLN- and harm (cons) of intervention for patients with pCLN+. There is also the harm (cons) of missed intervention for pCLN+ patients. Pros minus cons is the net benefit. When Pi < Pt, there is no intervention, and the net benefit is 0 (treat-none). When Pi > Pt, all patients receive the intervention, and the net benefit is shown by a gray slanted curve (treat-all). Our decision curve indicates that when the threshold probability is approximately 10% to 90%, the use of this predictive model would accrue greater benefit than a treat-all or treat-none strategy.

According to this model, the ROC curves of the derivation and validation groups were drawn, and the area under the curve (AUC) was 0.784 (95% CI 0.763-0.804) in the derivation group and 0.787 (95% CI 0.756-0.818) in the validation group. The C-indexes of the derivation and validation groups were both 0.783, which demonstrated good discriminative ability. The calibration plot revealed excellent agreement between the predictions and actual observations **(** **Figure 3** **)**. These results showed that this nomogram had good efficacy in predicting the probability of pCLN-. DCA was performed in the validation group. The results suggested that when the threshold probability was approximately 10% to 90%, compared to a “treat all” or “treat none” strategy, this nomogram yielded a greater net benefit, which indicated that this nomogram had good clinical value **(** **Figure 4** **)**.

### Follow-up Status

**Figure 5:**
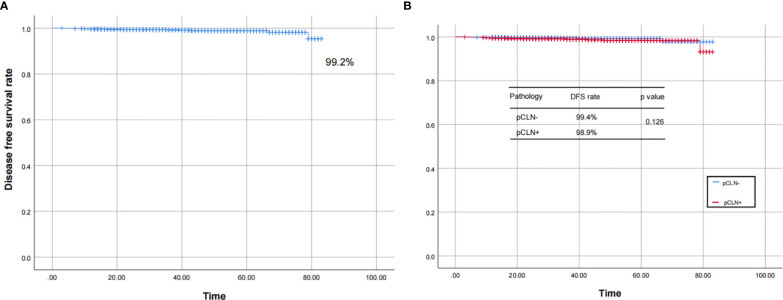
The Kaplan–Meier curves for the 2,497 patients. The curves show that the DFS rate was 99.2% in 2,497 patients **(A)**, and there was no significant difference in the DFS rate between the pCLN- and pCLN+ cohorts **(B)**.

We followed 2,497 patients (follow-up rate 92.9%) after the initial surgery until Dec. 2019. For patients without regular evaluations, DFS was based on the time of their last evaluation. The median follow-up period was 30 months (range 3–83 months). Among all 2,497 patients, 2,350 (94.1%) and 1,475 (59.1%) patients received TSH suppression and RAI therapy, respectively. Twenty-one (0.8%) patients experienced recurrence, and 3 (0.1%) died (1 due to asphyxia caused by tracheal metastasis and 2 due to other nontumorous causes). The survival curves plotted according to the Kaplan-Meier method showed that the DFS rate of the 2,497 patients was 99.2%. There was no significant difference in the DFS rate between the pCLN- and pCLN+ cohorts (99.4% *vs.* 98.9%, P=0.126) **(** **Figure 5** **)**. We also found significant differences in Tg levels and cervical ultrasound findings between recurrence and nonrecurrence patients (both P < 0.001), which revealed the importance of periodic monitoring of serum Tg levels and cervical ultrasound.(not shown in tables).

## Discussion

The present study indicated that for cN0 PTC, sex, age, TSH, size, location, unifocality, laterality, and ETE- were independent predictors of pCLN- but BMI and HT were not predictive.

The pCLN- cohorts had significantly lower CLNY and CLNR. This result was primarily because once the presence of CLNM was confirmed on intraoperative frozen section biopsy, prophylactic CLND was changed to therapeutic CLND, and the extent of CLND was further expanded.

The morbidity of PTC is higher in females than males, but the incidence of CLNM is lower (2, 18, 19). The present study supports these observations, and there was a clear tendency for females to present pCLN- (OR 2.069, P < 0.001). Age is an important basis for TNM staging, and it is one of the factors of many classic prognostic systems: AMES [age, metastasis (distance), extent, size], AGES (age, sex, extent, size), and MACIS [metastasis (distance), age, completeness of surgery, invasiveness, size], which suggests that age is closely related to the biological behavior of PTC. Prognosis and staging may be poorer with increasing age (6, 20, 21). We found that older patients (age > 45 years) generally had a significantly higher incidence of pCLN- (22, 23), especially patients over 65 years old, due to the susceptibility of older patients to lateral or distant metastasis (24, 25). Although no significant difference in pCLN- possibility was found among the three groups in young patients (age ≤ 45 years), patients aged 35 to 45 scored higher on the nomogram scale.

TSH regulates the proliferation and function of thyrocytes, and TSH suppression is an important aspect of the comprehensive treatment for PTC. Many studies showed that TSH was associated with tumorigenesis in PTC (26, 27). An increase in TSH may promote tumor progression, and a higher TSH level is a risk factor for PTC and lymph node metastasis (28, 29). Patients on levothyroxine or antithyroid drugs preoperatively were excluded from the present study, and we further divided the TSH plasma concentrations into 6 ranges. We found that in the 3 ranges of TSH ≤ 3 μIU/mL, the probability of PCLN- increased with decreasing TSH levels, and the opposite was true in the other 3 ranges of TSH > 3 μIU/mL. This result suggests that the level of TSH and the probability of PCLN- are not simple linear correlations.

Size reflects solid tumor staging, and it is related to many aspects, such as treatment and prognosis. PTC with a size ≤10 mm is defined as papillary thyroid microcarcinoma (PTMC), and most of these cases tend to exhibit less CLNM and a better prognosis, which suggests indolent behavior. Yu et al. included 11,845 PTMC patients in the SEER database and found that the LNM incidence of PTMC was 12%. Similarly, Ito et al. revealed an LNM incidence of <5% based on 5-10 years of PTMC data, and all new metastases appeared in the lateral compartment instead of the central compartment (5, 30, 31). Compared to PTC, which has an incidence range of 30-90%, PTMC is less prone to LN involvement, which suggests that PTC of a small size exhibits less nodal invasion (16, 32). We found that even for PTMC, compared to size > 5 but ≤ 10 mm, size ≤ 5 mm significantly increased the possibility of pCLN-, which obtained the highest score on the nomogram. Notably, the tumor size almost linearly negatively correlated with the probability of pCLN- except for the size > 25 but ≤ 30 mm group. The number of patients with tumor sizes between 25 and 30 mm in this study was the lowest(71 in total and 47 in the derivation group). More detailed studies are needed to determine whether this result was due to the decrease in CLNM when the tumor developed to a certain critical size or to the bias caused by an insufficient sample size.

Lymphatic vessels originating at the thyroid form a subcapsular lymphatic network on the surface of the gland. In addition to the superior and inferior thyroid arteries and veins, some lymphatic vessels run along the middle thyroid vein, and some do not accompany the blood vessels. This network is how thyroid-centered lymphatic vessels connect to the surrounding cervical lymph nodes. Different thyroid gland locations have different lymphatic drainage paths. Lymphatic drainage of the upper thyroid is primarily collected by the lymphatic vessels accompanying the superior thyroid artery, with some drainage flowing from the central to the lateral compartment, and some drainage flowing into the venous system through the lymph nodes of the lateral compartment. Lymphatic drainage of the lower thyroid is primarily collected by the lymphatic vessels accompanying the inferior thyroid artery and flowing into the lateral lymph nodes through the CLNs (especially the paratracheal lymph nodes) and ultimately into the venous system. Lymphatic drainage from the isthmus of the thyroid primarily descends to the mediastinal lymph nodes (33, 34). We found that the middle portion was the most common location for PTC (43.7%) and the location with the highest likelihood of pCLN-. This result may be explained by the more complex and crisscrossing lymphatic drainage pathways in the middle portion, and drainage of this region may be carried up or down before proceeding through the upper or lower lymphatic vessels to the corresponding lymph nodes. Therefore, middle tumors progress more slowly than tumors in other portions. There is also a lateral drainage pathway in the middle of the thyroid that passes through the deep surface of the cervical sheath. However, these hypotheses emphasize the need for more accurate anatomical studies to understand the patterns of metastatic spread in PTC. Previous studies reported that an upper location was a risk factor for LLNM, and tumors located in the isthmus and lower portion were more likely to result in CLNM (35, 36). Consistent with these studies, we found that the probability of pCLN- in upper tumors was second only to middle tumors. The probability of pCLN- in isthmic and lower tumors was reduced even further.

Bilaterality or multifocality may develop *via* intraglandular spread from a single primary tumor or multiple synchronous primary tumors (37). The invasiveness of unilateral and unifocal PTC is weaker than bilateral and multifocal PTC (38, 39). The present study also suggested that CLNs were less prone to invasion in unilateral or unifocal PTC. We further found no significant difference in the probability of pCLN- between the left and right lobes. The left and right lobes of the thyroid gland were not completely symmetrical, but the right lobe was generally larger (40).

ETE- refers to tumors confined to the thyroid capsule. It is easier for the tumor to invade lymph nodes through lymphatics on the surface of the capsule after the thyroid capsule is breached (4, 41). We also found that ETE- was separately and independently associated with pCLN- in cN0 PTC.

The incidence of being overweight increased synchronously with the morbidity of PTC (42), but the relationship between BMI and PTC is not clear. A study of 11,323,006 adults showed that BMI and waist circumference were associated with PTC, and weight loss was associated with lower PTC morbidity (43). Kang et al. and Ray et al. found that fibroblast growth factor 21 (FGF21) positively correlated with BMI, and leptin played a role as a proinflammatory factor in overweight people. FGF21 and leptin were also involved in the regulation of PTC development, which indicated that BMI was also related to invasion and metastasis in PTC (44, 45). The BMI level of the pCLN- cohort in the present study was slightly lower, but there was no significant correlation between BMI and pCLN-, which is consistent with the results obtained by Grani et al. and Paes et al. (46, 47). Because being overweight may involve aromatase activation, chronic inflammation and oxidative stress, more molecular biological mechanisms that affect the relationship between BMI, PTC and invasiveness must be explored.

HT is a type of autoimmune thyroiditis that is characterized by an abundance of lymphocytes, and it also known as chronic lymphocyte thyroiditis. The relationship between HT and CLN status in PTC is inconclusive (48, 49), and the differences in diagnostic criteria, severity, antibody levels, and the heterogeneity of HT may underlie these controversies. HT was confirmed *via* postoperative pathology in this study, and we found that HT barely affected CLN status. However, this study did not determine the level of autoantibodies (primarily TPO-Ab and Tg-Ab), and quantitative analysis of the degree of HT before surgery needs further study.

The follow-up results showed that very few patients experienced lymph node recurrence, which confirmed that the lymph node pathologic status, especially in the pCLN- cohort was reliable. Specifically, the derivation basis of the nomogram was reliable. Because the present study was a single-center retrospective study, selection deviation is inevitable. Notably, the lack of external validation is a limitation, and relevant prospective multicenter clinical studies should be performed in the future to evaluate the accuracy of the proposed model. Analysis of potential confounders of future metastasis was not possible due to the relatively short follow-up, which suggests that prolonged follow-up and more data are required in our further work.

There have been many prediction models of CLNM in cN0 PTC (50, 51). However, available prediction models for other patients who present pCLN- and constitute the majority of the study population (the pCLN- rate was 51.5% in this study) are lacking. In conclusion, we established the first quantified prediction model for pCLN- in cN0 PTC. Clinicians can use this nomogram to evaluate patients with PTC based on clinicopathological characteristics. For cN0 PTC, which has a high probability of pCLN-, unnecessary prophylactic CLND can may be avoided.

## Data Availability Statement

The original contributions presented in the study are included in the article/supplementary material. Further inquiries can be directed to the corresponding author.

## Ethics Statement

The studies involving human participants were reviewed and approved by Ethics Committee of the First Affiliated Hospital of Chongqing Medical University. Written informed consent for participation was not required for this study in accordance with the national legislation and the institutional requirements.

## Author Contributions

XJS and XLS designed this study. XJS, LT, DH, YW, PY, ZY, CD, and DW collected the data. XJS analyzed the data. All authors contributed to the article and approved the submitted version.

## Conflict of Interest

The authors declare that the research was conducted in the absence of any commercial or financial relationships that could be construed as a potential conflict of interest.

## Publisher’s Note

All claims expressed in this article are solely those of the authors and do not necessarily represent those of their affiliated organizations, or those of the publisher, the editors and the reviewers. Any product that may be evaluated in this article, or claim that may be made by its manufacturer, is not guaranteed or endorsed by the publisher.

## Abbreviations

cN0, clinical lymph node negativity; pN0, pathologic lymph node negativity; pCLN-, pathologic central lymph node negativity; PTC, papillary thyroid carcinoma; PTMC, papillary thyroid microcarcinoma; CLN, central lymph node; CLNM, central lymph node metastasis; CLND, central lymph node dissection; SET, scarless endoscopic thyroidectomy; BMI, body mass index; TSH, thyroid stimulating hormone; Tg, thyroglobulin; HT, Hashimoto’s thyroiditis; ETE-, extrathyroidal extension negativity (tumor confined to the thyroid); IQR, interquartile range; CLNY,central lymph node yield; CLNR,central lymph node ratio; AUC, area under curve; DFS, disease-free survival; OR, odd ratio; CI, confidence index.
